# Supplementary material for: Testing Analytical Methods for Antibiotic Detection in Tenebrio molitor Larvae: A Controlled Feeding Trial
Source: Antibiotics (Basel). 2025 Sep 9;14(9):909. doi: 10.3390/antibiotics14090909 (PMC12466411; doi:10.3390/antibiotics14090909)
Supplement: Supplementary file 1 [file antibiotics-14-00909-s001.zip › Supplementary material S1 Antibiotic detection in food.pdf]

Supplementing material S1: Test procedure for the determination of antibiotics in animal-origin foods (excluding honey) using LC-MS/MS

Chemicals / Materials

- Acetonitrile, HPLC grade (Merck, Darmstadt, Germany)
- Methanol, HPLC grade (Merck, Darmstadt, Germany)
- Deionized water
- Ultrapure water
- Formic acid (99%), Optima LC/MS (Thermo Fisher Scientific, Waltham, USA)
- Phosphoric acid, concentrated (Merck, Darmstadt, Germany)
- Citric acid x H_2O (Merck, Darmstadt, Germany)
- Na_2HPO_4 x 2 H_2O (Merck, Darmstadt, Germany)
- Titriplex III (Na_2EDTA x 2 H_2O) (Carl Roth, Karlsruhe, Germany)

Solid Phase Extraction (SPE) Cartridges: OASIS HLB (200 mg, 6 mL) (Waters Corporation, Milford, USA)

Solutions

- EMI Buffer: Weigh 31.5 g citric acid, 35.6 g Na_2HPO_4 x 2 H_2O, and 93 g Titriplex III, dissolve in deionized water, adjust pH to 4 with concentrated phosphoric acid, and make up to 2.5 L.
- Eluent A: 0.1% formic acid in ultrapure water (1 mL formic acid (98%) / 1000 mL)
- Eluent B: 0.1% formic acid in acetonitrile (1 mL formic acid (98%) / 1000 mL)

Standard Substances

Tiamulin (Merck, Darmstadt, Germany), Chloramphenicol (Merck, Darmstadt, Germany), Erythromycin (Merck, Darmstadt, Germany).

Preparation of Standard Solutions

Tiamulin and Erythromycin:

The standard stock solutions are each prepared at a concentration of 1 mg/ml in methanol.

Chloramphenicol:

The standard stock solution is prepared at a concentration of 100 µg/ml in methanol. The solutions are stable for 1 year at refrigerator temperature.

Mixed spiking solution

The mixed solution is prepared from the stock solutions by mixing and diluting with ultrapure water/methanol (90/10, v/v) as follows:

**Substance**	**(50 ml)**	
	**Volume** [µl] (Stock solution)	**Concentration** [ng/ml] (Substance in mixed solution)
Chloramphenicol	10	20
Tiamulin	50	1000
Erythromycin	100	2000

Procedure

Sample Preparation

1. Weigh 1.0 g of the ground and homogenized sample into a 50 mL centrifuge tube.

Extraction

1. Add 10 mL EMI buffer, vortex for 1 min, shake for 10 min, and sonicate for 5 min.

2. Centrifuge at 4000 rpm for 10 min.

3. Filter the supernatant and proceed with SPE purification.

SPE Purification

1. Condition an OASIS HLB SPE cartridge with 6 mL methanol and 6 mL deionized water.

2. Load the extraction solution onto the column.

3. Wash with 6 mL ultrapure water/methanol (95/5, v/v).

4. Elute with 6 mL methanol and evaporate to dryness.

5. Reconstitute in 1 mL ultrapure water/acetonitrile (90/10, v/v) and proceed with LC-MS analysis.

LC-MS Analysis

<u>Chromatographic Conditions</u>
- Flow rate: 0.3 mL/min
- Oven temperature: 30°C

- Column: Phenomenex Luna Omega, 1.6 µm, Polar C18, 2.1 mm x 100 mm with pre-column
- Injection volume: 20 µL
- Eluent A: 0,1 % formic acid in ultrapure water
- Eluent B: 0,1 % formic acid in acetonitrile

Gradient conditions:

**Time (min)**	**Flow Rate (ml/min)**	**Eluent A (%)**	**Eluent B (%)**
0	0,3	90	10
1	0,3	90	10
12	0,3	40	60
14	0,3	40	60
15	0,3	90	10
16	0,3	90	10

Post time: 5 min

MS

Ion source: ESI+Agilent Jetstream

Scan Typ: DynamicMRM

**Substance**	**MS-mass**	**MS/MS-mass**	**Polarity**
Tiamulin	495	192/119	positive
Erythromycin	734	158/83	positive
Chloramphenicol	321	257/152/121	Negativ

Quality control / matrix calibration

As a quality assurance measure accompanying the analysis, at least four additional tests are prepared for each series to check the recovery of the active substance. Four blank samples are spiked with 10µl, 50µl, 100µl and 150µl of the mixed spiking solution respectively and analysed.

These additional tests correspond to the following contents in µg/kg:

**Substance**	**additive 1**	**additive 2**	**additive 3**	**additive 4**
Tiamulin	10	50	100	150
Erythromycin	20	100	200	300
Chloramphenicol	0,2	1	2	3

Evaluation

The detection of a target analyte in a sample is carried out via the registered mass spectrometric data (usually mass traces of two MS2 daughter masses) and via the retention time by comparison with the corresponding values of a standard.

Quantification

A batch of each sample is first prepared and analysed. If a target analyte is detected, the analyte content is determined from the corresponding response using the matrix calibration function (created using the control samples).

In the case of a critical initial finding, several preparations of the sample are then reprocessed and analysed. To create the matrix calibration function, blank samples (e.g. five batches) are spiked with different amounts of analyte, processed and analysed. The individual concentration levels of the analyte to be analysed and the internal standard are determined by the test supervisor. The final result is obtained by averaging.
