# Supplementary material for: Testing Analytical Methods for Antibiotic Detection in Tenebrio molitor Larvae: A Controlled Feeding Trial
Source: Antibiotics (Basel). 2025 Sep 9;14(9):909. doi: 10.3390/antibiotics14090909 (PMC12466411; doi:10.3390/antibiotics14090909)
Supplement: Supplementary file 1 [file antibiotics-14-00909-s001.zip › Supplementary material S3 LC_MS_MS_rawdata.pdf]

Supplementary material S 3: LC–MS/MS raw data

Supplement to result section 2.2. Evaluation of homogeneity and stability of antibiotic-containing feed samples using LC-MS/MS analysis

Tiamulin

Sample IDs Tiamulin:

240337170-1 to -5: Homogentiy

240337172: control; 240337173, 240337174: Stability

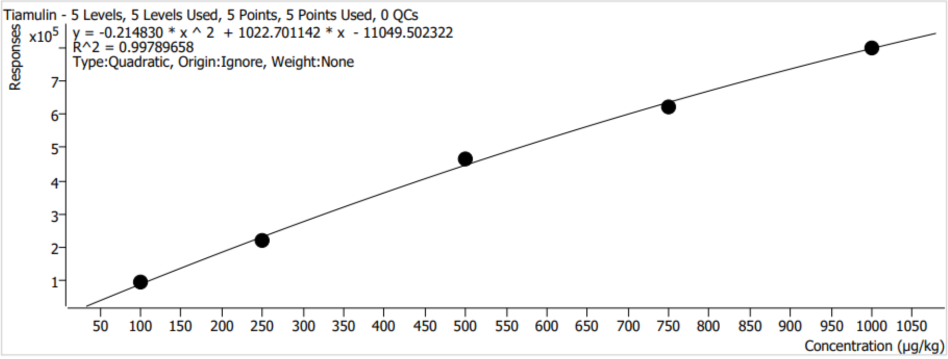

Quantifier

Data File	Sample Name	Sample Type	RT	Compound	Area	Final Conc	Unit
Multi-FM24-06-26-03.d	Multi Mix FM	Sample	7.873	Tiamulin	2320341	ND	µg/kg
Multi-FM24-06-26-04.d	LW+1000µg/kg	Cal	7.773	Tiamulin	800694	1006.5441	µg/kg
Multi-FM24-06-26-05.d	LW+750µg/kg	Cal	7.773	Tiamulin	621530	730.6920	µg/kg
Multi-FM24-06-26-06.d	LW+500µg/kg	Cal	7.798	Tiamulin	464674	522.5152	µg/kg
Multi-FM24-06-26-07.d	LW+250µg/kg	Cal	7.789	Tiamulin	218707	236.3951	µg/kg
Multi-FM24-06-26-08.d	LW+100µg/kg	Cal	7.773	Tiamulin	93217	104.2341	µg/kg
Multi-FM24-06-26-09.d	LW	Sample	7.831	Tiamulin	22	10.8501	µg/kg
Multi-FM24-06-26-10.d	240337170 -1	Sample	7.865	Tiamulin	1237272	ND	µg/kg
Multi-FM24-06-26-11.d	240337170 -2	Sample	7.865	Tiamulin	1063405	1565.2665	µg/kg
Multi-FM24-06-26-12.d	240337170 -3	Sample	7.865	Tiamulin	1392790	ND	µg/kg
Multi-FM24-06-26-13.d	240337170 -4	Sample	7.856	Tiamulin	1513855	ND	µg/kg
Multi-FM24-06-26-14.d	240337170 -5	Sample	7.865	Tiamulin	1360674	ND	µg/kg
Multi-FM24-06-26-15.d	240337172	Sample	7.873	Tiamulin	211	11.0365	µg/kg
Multi-FM24-06-26-16.d	240337173	Sample	7.865	Tiamulin	871216	1131.7312	µg/kg
Multi-FM24-06-26-17.d	240337174	Sample	7.865	Tiamulin	952673	1294.1404	µg/kg
Multi-FM24-06-26-18.d	LW+500µg/kg	Sample	7.798	Tiamulin	344634	377.7659	µg/kg
Multi-FM24-06-26-19.d	Multi Mix FM	Sample	7.873	Tiamulin	1661749	ND	µg/kg

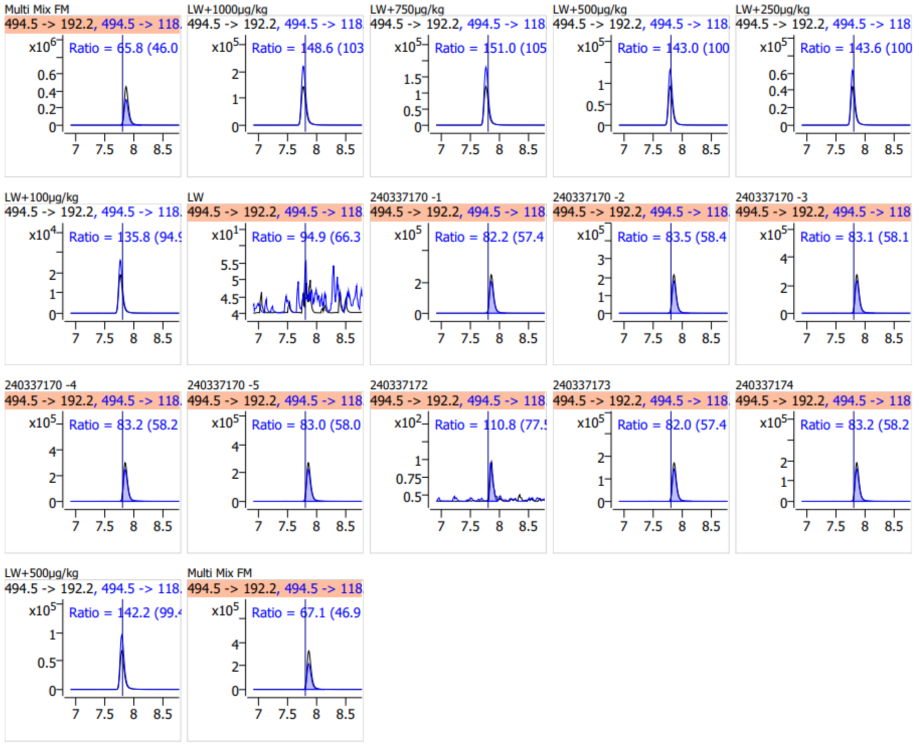

Matrix calibration Tiamulin

x	y	b
0,000001		30213,6585
100	93217	787,59758
250	218707	
500	464674	
750	621530	
1000	800694	

Chloramphenicol

Sample IDs Chloramphenicol:

240382883 a to e: Homogenity
240382886, 240382887, 240382888: Stability

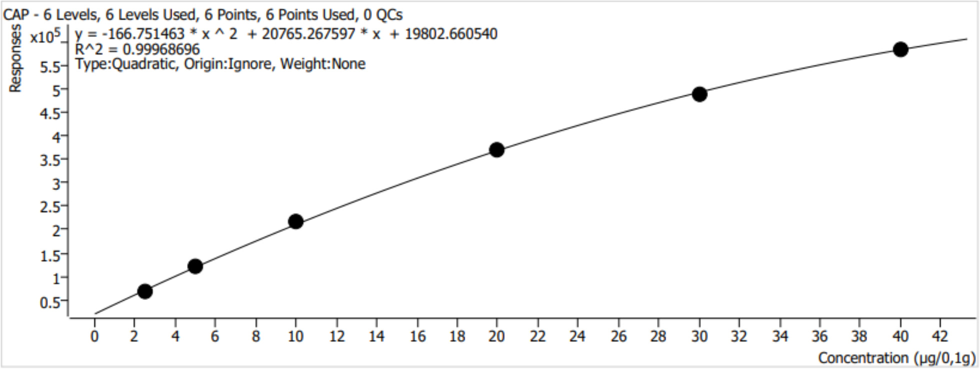

Quantifier

Data File	Sample Name	Sample Type	RT	Compound	Area	Final Conc	Unit
CAP-FM24-08-28-03.d	CAP Mix	Sample	10.147	CAP	538	0.0000	µg/0,1g
CAP-FM24-08-28-04.d	LW+40µg/0.1g	Cal	10.147	CAP	585716	40.2853	µg/0,1g
CAP-FM24-08-28-05.d	LW+30µg/0.1g	Cal	10.147	CAP	488479	29.6115	µg/0,1g
CAP-FM24-08-28-06.d	LW+20µg/0.1g	Cal	10.147	CAP	368253	19.9890	µg/0,1g
CAP-FM24-08-28-07.d	LW+10µg/0.1g	Cal	10.147	CAP	215312	10.2607	µg/0,1g
CAP-FM24-08-28-08.d	LW+5µg/0.1g	Cal	10.147	CAP	121644	5.1145	µg/0,1g
CAP-FM24-08-28-09.d	LW+2.5µg/0.1g	Cal	10.147	CAP	66214	2.2767	µg/0,1g
CAP-FM24-08-28-10.d	LW	Sample	10.266	CAP	5	0.0000	µg/0,1g
CAP-FM24-08-28-11.d	240382883 a	Sample	10.147	CAP	213633	10.1640	µg/0,1g
CAP-FM24-08-28-12.d	240382883 b	Sample	10.171	CAP	216564	10.3329	µg/0,1g
CAP-FM24-08-28-13.d	240382883 c	Sample	10.171	CAP	182562	8.4054	µg/0,1g
CAP-FM24-08-28-14.d	240382883 d	Sample	10.171	CAP	201272	9.4573	µg/0,1g
CAP-FM24-08-28-15.d	240382883 e	Sample	10.171	CAP	384229	21.1378	µg/0,1g
CAP-FM24-08-28-16.d	LW	Sample	10.052	CAP	6	0.0000	µg/0,1g
CAP-FM24-08-28-17.d	240382886	Sample	10.171	CAP	229452	11.0825	µg/0,1g
CAP-FM24-08-28-18.d	240382887	Sample	10.171	CAP	228482	11.0256	µg/0,1g
CAP-FM24-08-28-19.d	LW	Sample	10.005	CAP	10	0.0000	µg/0,1g
CAP-FM24-08-28-20.d	240382888	Sample	10.171	CAP	3	0.0000	µg/0,1g
CAP-FM24-08-28-21.d	LW+10µg/0.1g	Sample	10.171	CAP	234537	11.3812	µg/0,1g
CAP-FM24-08-28-22.d	CAP Mix	Sample	10.171	CAP	638	0.0000	µg/0,1g

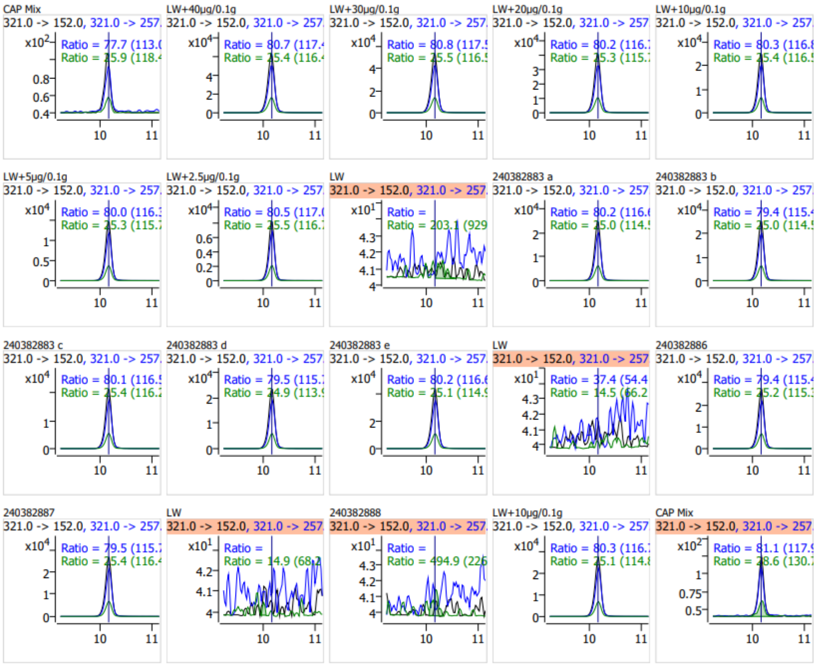

Erythromycin

Sample IDs Erythromycin:

240382858 a to e: Homogenity

240382860, 240382861 , 240382862: Stability

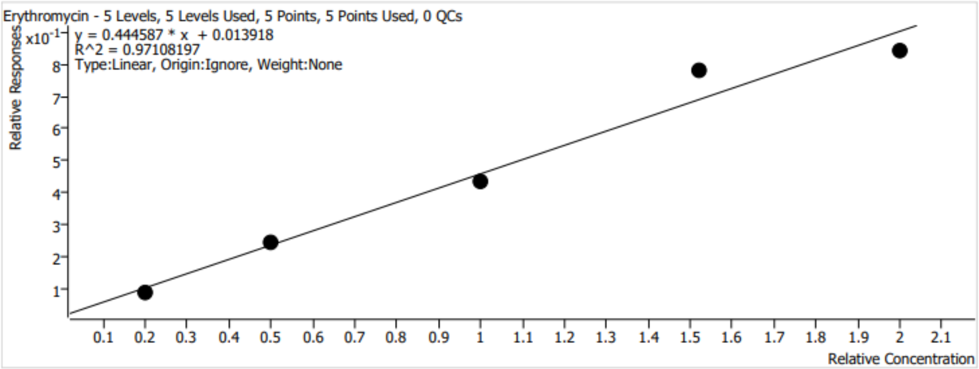

Quantifier

Data File	Sample Name	Compound	RT	Area	Final Conc	Unit	ISTD	ISTD RT	ISTD Area
Multi-FM24-08-09-03.d	Multi-FM Mix	Erythromycin	7.051	208018	296.8789	µg/g	Roxithromycin	8.422	383887
Multi-FM24-08-09-04.d	LW+500µg/g	Erythromycin	7.042	1231096	467.5455	µg/g	Roxithromycin	8.413	1456269
Multi-FM24-08-09-05.d	LW+380µg/g	Erythromycin	7.051	1144664	431.5919	µg/g	Roxithromycin	8.422	1464816
Multi-FM24-08-09-06.d	LW+250µg/g	Erythromycin	7.051	646386	236.4469	µg/g	Roxithromycin	8.422	1487987
Multi-FM24-08-09-07.d	LW+125µg/g	Erythromycin	7.059	355992	128.8643	µg/g	Roxithromycin	8.430	1464481
Multi-FM24-08-09-08.d	LW+50µg/g	Erythromycin	7.059	121061	40.5514	µg/g	Roxithromycin	8.422	1407150
Multi-FM24-08-09-09.d	LW	Erythromycin	7.051	1732	0.0000	µg/g	Roxithromycin	8.430	1343011
Multi-FM24-08-09-10.d	240382858 a	Erythromycin	7.059	452253	190.5951	µg/g	Roxithromycin	8.430	1281668
Multi-FM24-08-09-11.d	240382858 b	Erythromycin	7.059	492018	185.5733	µg/g	Roxithromycin	8.430	1430569
Multi-FM24-08-09-12.d	240382858 c	Erythromycin	7.059	378091	166.9723	µg/g	Roxithromycin	8.430	1216301
Multi-FM24-08-09-13.d	240382858 d	Erythromycin	7.059	329490	144.1751	µg/g	Roxithromycin	8.430	1218927
Multi-FM24-08-09-14.d	240382858 e	Erythromycin	7.059	526376	241.1652	µg/g	Roxithromycin	8.430	1188760
Multi-FM24-08-09-15.d	240382860	Erythromycin	7.059	474646	241.6853	µg/g	Roxithromycin	8.430	1069699
Multi-FM24-08-09-16.d	240382861	Erythromycin	7.059	336356	185.9439	µg/g	Roxithromycin	8.430	976100
Multi-FM24-08-09-17.d	LW	Erythromycin	7.051	1453	0.0000	µg/g	Roxithromycin	8.430	1029670
Multi-FM24-08-09-18.d	240382862	Erythromycin	7.067	1244	0.0000	µg/g	Roxithromycin	8.430	958295
Multi-FM24-08-09-19.d	LW+250µg/g	Erythromycin	7.059	488010	264.7750	µg/g	Roxithromycin	8.430	1006662
Multi-FM24-08-09-20.d	Multi-FM Mix	Erythromycin	7.059	168293	355.8396	µg/g	Roxithromycin	8.438	260223

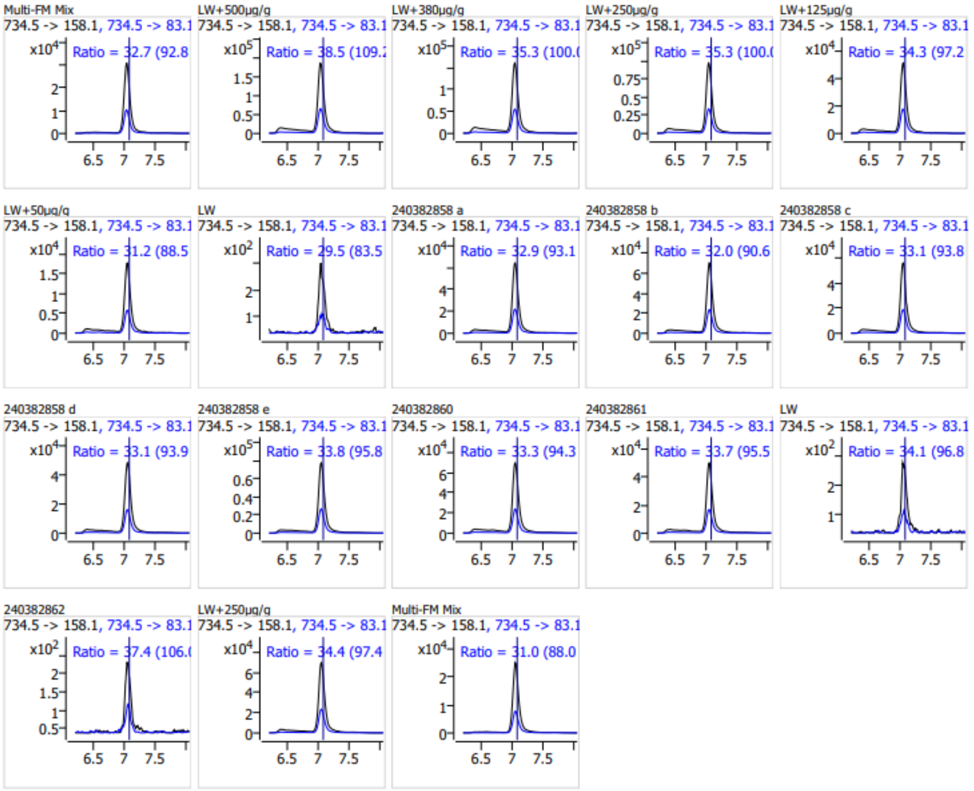

Matrix calibration Chloramphenicol & Erythromycin

	Chloramphenicol / µg/kg	Erythromycin / µg/kg
Z1	100	5000
Z2	500	10000
Z3	1000	15000
Z4	2000	20000
Z5	3000	25000
Z6	5000	30000

Supplement to result section 2.3. Detection and Quantification of antibiotics in mealworms using LC-MS/MS analysis

Tiamulin screening

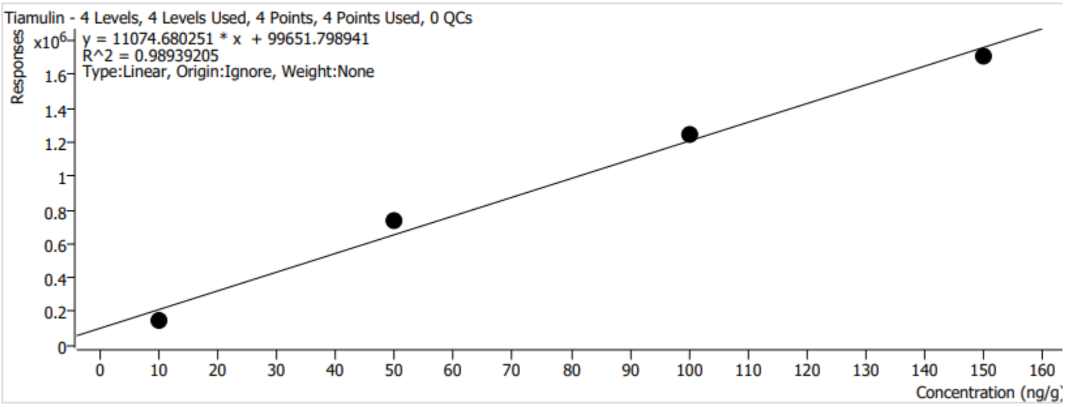

Quantifier

Data File	Sample Name	Sample Type	RT	Compound	Area	Final Conc	Unit
Multi24-07-18-01.d	Multi Mix Muskel	Sample	9.145	Tiamulin	6900064	614.0504	ng/g
Multi24-07-18-02.d	LW+Z4 Tiamulin	Cal	9.145	Tiamulin	1714661	145.8290	ng/g
Multi24-07-18-03.d	LW+Z3 Tiamulin	Cal	9.137	Tiamulin	1243861	103.3176	ng/g
Multi24-07-18-04.d	LW+Z2 Tiamulin	Cal	9.145	Tiamulin	732393	57.1340	ng/g
Multi24-07-18-05.d	LW+Z1 Tiamulin	Cal	9.137	Tiamulin	140843	3.7194	ng/g
Multi24-07-18-06.d	LW	Sample	9.145	Tiamulin	211	0.0000	ng/g
Multi24-07-18-07.d	240337200	Sample	9.179	Tiamulin	39	0.0000	ng/g
Multi24-07-18-08.d	240337201	Sample	9.078	Tiamulin	22408449	2014.3965	ng/g
Multi24-07-18-09.d	240337202	Sample	9.137	Tiamulin	4167959	367.3522	ng/g
Multi24-07-18-10.d	LW QC	Sample	9.137	Tiamulin	4608	0.0000	ng/g
Multi24-07-18-11.d	LW QC+Z3 Tiamulin	Sample	9.137	Tiamulin	1153314	95.1416	ng/g
Multi24-07-18-12.d	Multi Mix Muskel	Sample	9.154	Tiamulin	6162107	547.4158	ng/g

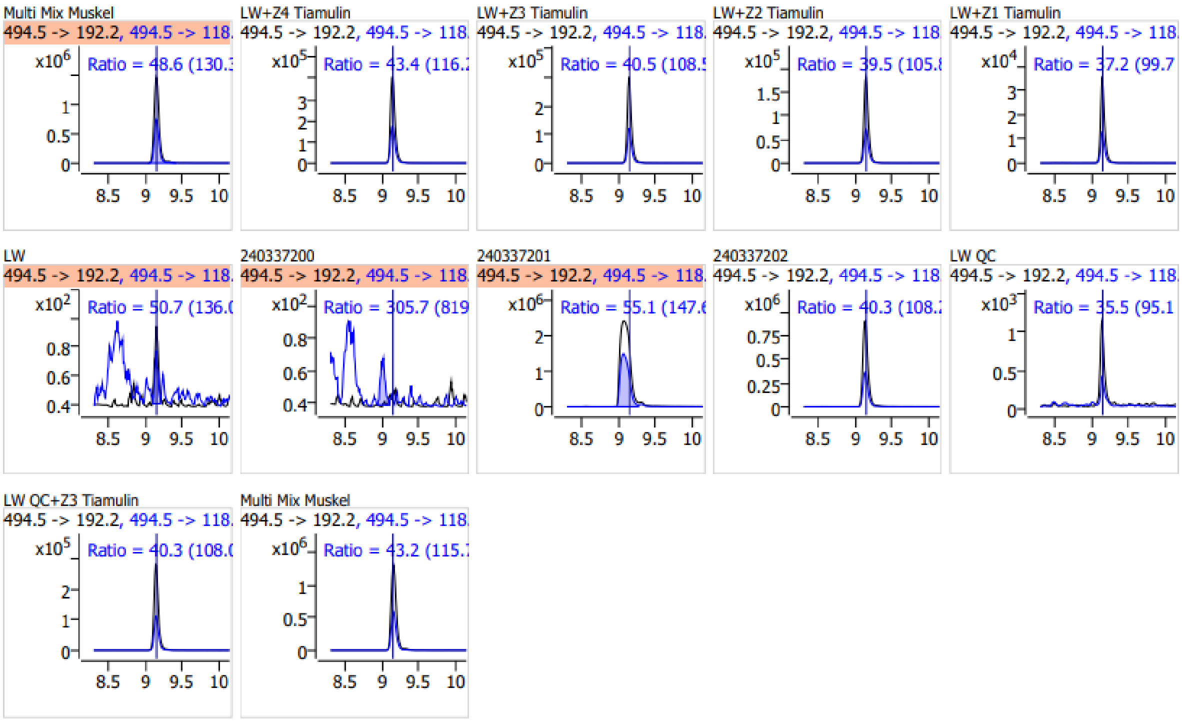

Tiamulin quantification in meal worms after 0 h of fasting:

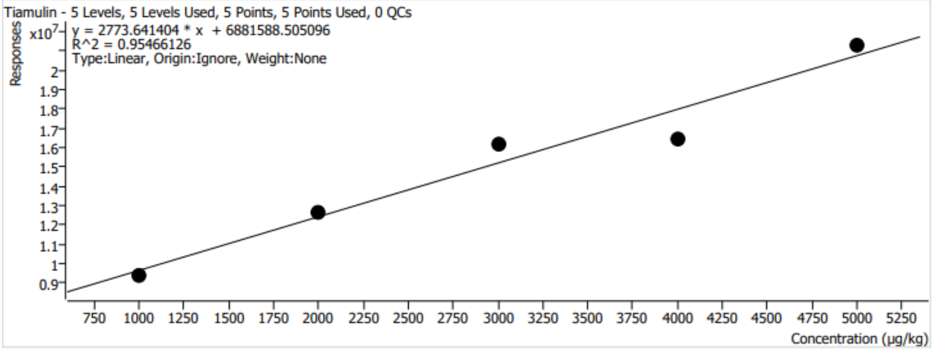

Quantifier

Data File	Sample Name	Sample Type	RT	Compound	Area	Final Conc	Unit
Multi24-07-31-03.d	Multi Mix	Sample	9.154	Tiamulin	6431903	0.0000	µg/kg
Multi24-07-31-04.d	Mehlwurm LW+5000µg/kg	Cal	9.103	Tiamulin	21326271	5207.8408	µg/kg
Multi24-07-31-05.d	Mehlwurm LW+4000µg/kg	Cal	9.112	Tiamulin	16480196	3460.6520	µg/kg
Multi24-07-31-06.d	Mehlwurm LW+3000µg/kg	Cal	9.112	Tiamulin	16168029	3348.1042	µg/kg
Multi24-07-31-07.d	Mehlwurm LW+2000µg/kg	Cal	9.129	Tiamulin	12679809	2090.4723	µg/kg
Multi24-07-31-08.d	Mehlwurm LW+1000µg/kg	Cal	9.137	Tiamulin	9358258	892.9307	µg/kg
Multi24-07-31-09.d	Mehlwurm LW+500µg/kg	Sample	9.145	Tiamulin	5455890	0.0000	µg/kg
Multi24-07-31-10.d	Mehlwurm LW+400µg/kg	Sample	9.145	Tiamulin	4794223	0.0000	µg/kg
Multi24-07-31-11.d	Mehlwurm LW+300µg/kg	Sample	9.154	Tiamulin	4241645	0.0000	µg/kg
Multi24-07-31-12.d	Mehlwurm LW+200µg/kg	Sample	9.162	Tiamulin	3137433	0.0000	µg/kg
Multi24-07-31-13.d	Mehlwurm LW+100µg/kg	Sample	9.162	Tiamulin	1595919	0.0000	µg/kg
Multi24-07-31-14.d	Mehlwurm LW	Sample	9.162	Tiamulin	4920	0.0000	µg/kg
Multi24-07-31-15.d	240337201 a	Sample	9.103	Tiamulin	25089261	6564.5372	µg/kg
Multi24-07-31-16.d	240337201 b	Sample	9.103	Tiamulin	26541401	7088.0874	µg/kg
Multi24-07-31-17.d	240337201 c	Sample	9.103	Tiamulin	25856398	6841.1183	µg/kg
Multi24-07-31-18.d	Mehlwurm LW	Sample	9.162	Tiamulin	10037	0.0000	µg/kg
Multi24-07-31-19.d	240337202 a	Sample	9.154	Tiamulin	6243731	0.0000	µg/kg
Multi24-07-31-20.d	240337202 b	Sample	9.154	Tiamulin	6342188	0.0000	µg/kg
Multi24-07-31-21.d	240337202 c	Sample	9.162	Tiamulin	5892374	0.0000	µg/kg
Multi24-07-31-22.d	Mehlwurm LW+1000µg/kg	Sample	9.145	Tiamulin	9377123	899.7323	µg/kg
Multi24-07-31-23.d	Multi Mix	Sample	9.171	Tiamulin	5724570	0.0000	µg/kg

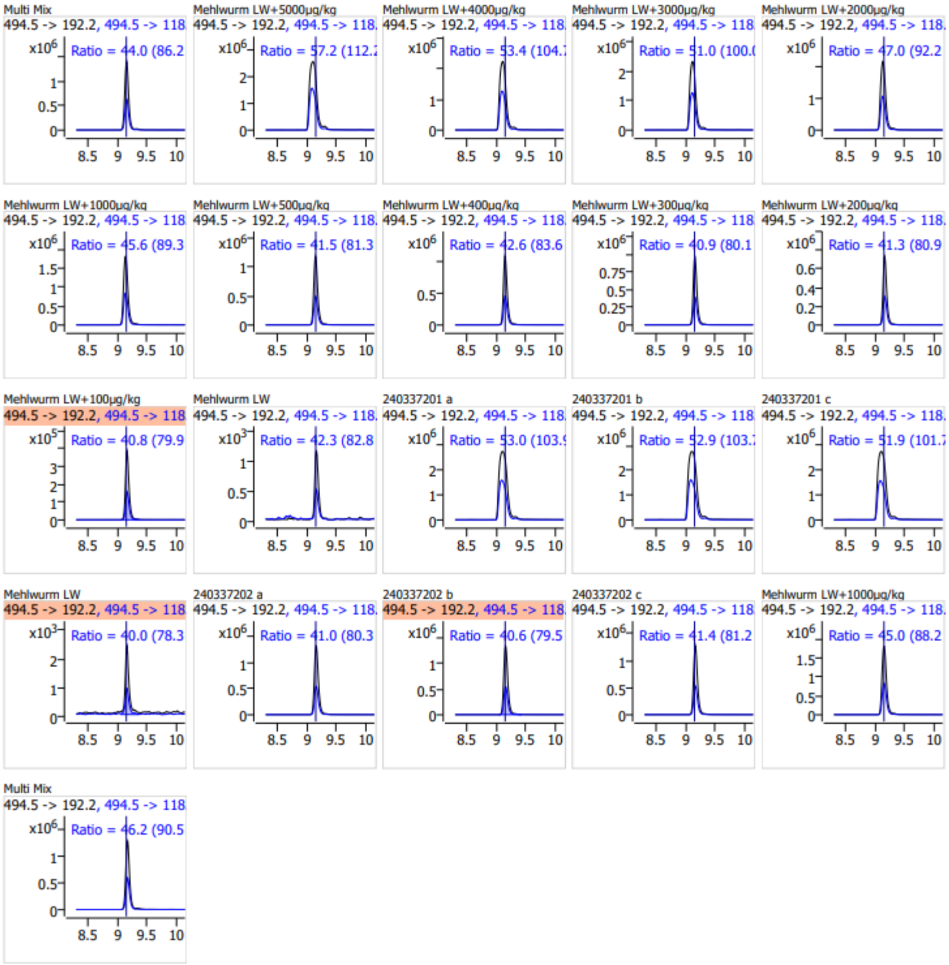

Tiamulin quantification in meal worms after 24 h of fasting:

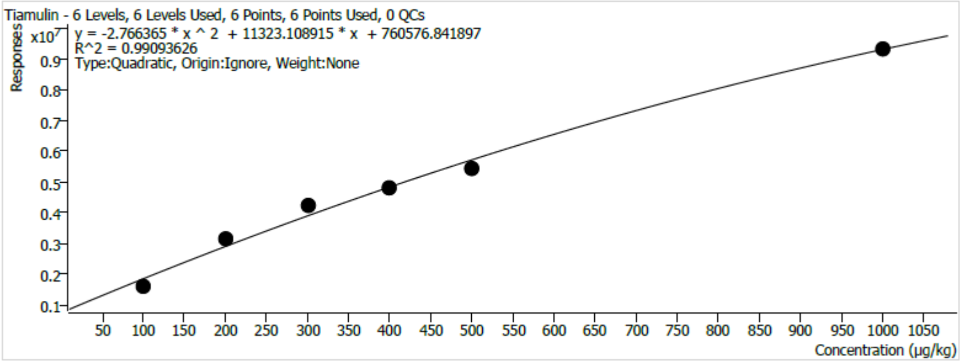

Data File	Sample Name	Sample Type	RT	Compound	Area	Final Conc	Unit
Multi24-07-31-03.d	Multi Mix	Sample	9.154	Tiamulin	6431903	584.2615	µg/kg
Multi24-07-31-04.d	Mehlwurm LW+5000µg/kg	Sample	9.103	Tiamulin	21326271	ND	µg/kg
Multi24-07-31-05.d	Mehlwurm LW+4000µg/kg	Sample	9.112	Tiamulin	16480196	ND	µg/kg
Multi24-07-31-06.d	Mehlwurm LW+3000µg/kg	Sample	9.112	Tiamulin	16168029	ND	µg/kg
Multi24-07-31-07.d	Mehlwurm LW+2000µg/kg	Sample	9.129	Tiamulin	12679809	ND	µg/kg
Multi24-07-31-08.d	Mehlwurm LW+1000µg/kg	Cal	9.137	Tiamulin	9358258	1007.0939	µg/kg
Multi24-07-31-09.d	Mehlwurm LW+500µg/kg	Cal	9.145	Tiamulin	5455890	468.2288	µg/kg
Multi24-07-31-10.d	Mehlwurm LW+400µg/kg	Cal	9.145	Tiamulin	4794223	394.1947	µg/kg
Multi24-07-31-11.d	Mehlwurm LW+300µg/kg	Cal	9.154	Tiamulin	4241645	334.8186	µg/kg
Multi24-07-31-12.d	Mehlwurm LW+200µg/kg	Cal	9.162	Tiamulin	3137433	221.9469	µg/kg
Multi24-07-31-13.d	Mehlwurm LW+100µg/kg	Cal	9.162	Tiamulin	1595919	75.1531	µg/kg
Multi24-07-31-14.d	Mehlwurm LW	Sample	9.162	Tiamulin	4920	0.0000	µg/kg
Multi24-07-31-15.d	240337201 a	Sample	9.103	Tiamulin	25089261	ND	µg/kg
Multi24-07-31-16.d	240337201 b	Sample	9.103	Tiamulin	26541401	ND	µg/kg
Multi24-07-31-17.d	240337201 c	Sample	9.103	Tiamulin	25856398	ND	µg/kg
Multi24-07-31-18.d	Mehlwurm LW	Sample	9.162	Tiamulin	10037	0.0000	µg/kg
Multi24-07-31-19.d	240337202 a	Sample	9.154	Tiamulin	6243731	561.1854	µg/kg
Multi24-07-31-20.d	240337202 b	Sample	9.154	Tiamulin	6342188	573.2144	µg/kg
Multi24-07-31-21.d	240337202 c	Sample	9.162	Tiamulin	5892374	519.0300	µg/kg
Multi24-07-31-22.d	Mehlwurm LW+1000µg/kg	Sample	9.145	Tiamulin	9377123	1010.3793	µg/kg
Multi24-07-31-23.d	Multi Mix	Sample	9.171	Tiamulin	5724570	499.3024	µg/kg

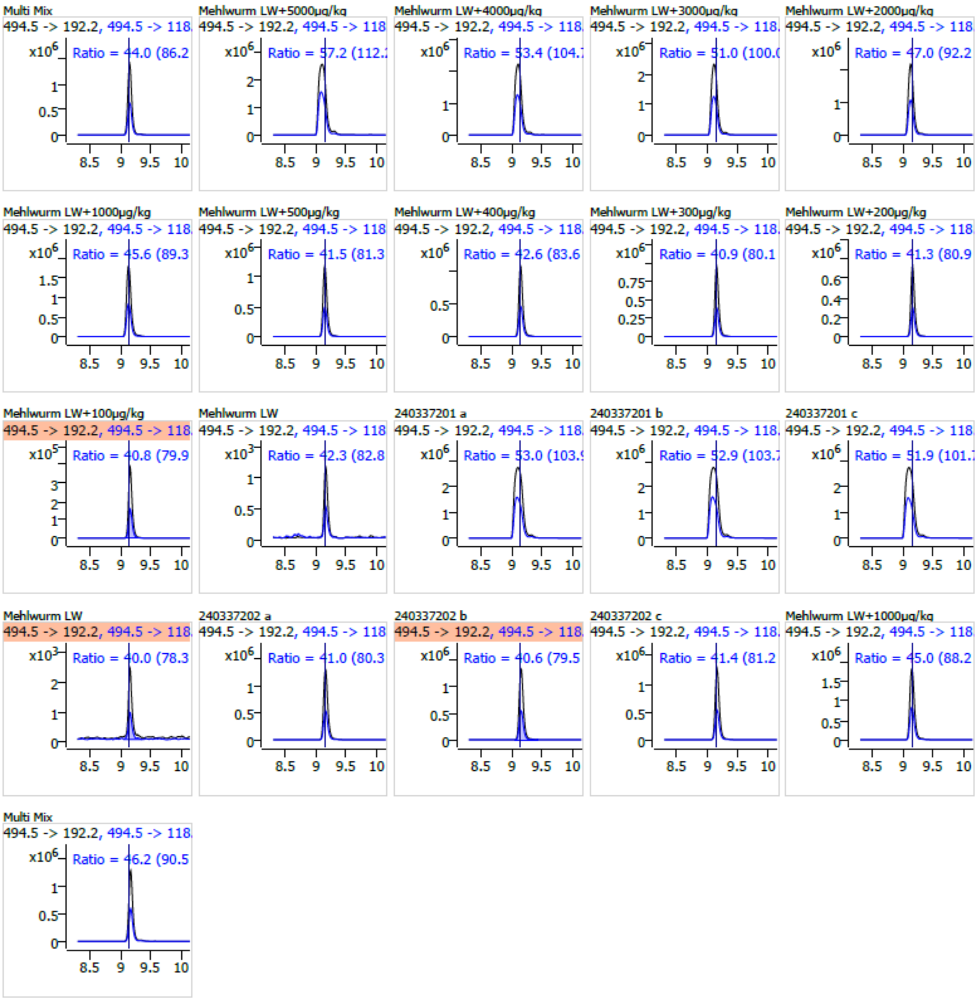

Chloramphenicol quantification in meal worms

Sample IDs Chloramphenicol:

240382889: Meal worms after 0 h fasting
240382890: Meal worms after 24h fasting
240382891: Control

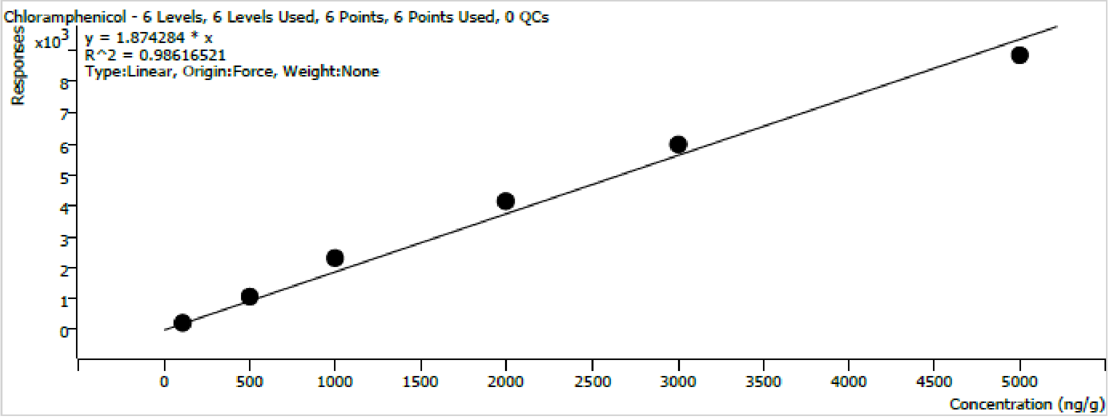

Quantifier

Data File	Sample Name	Sample Type	RT	Compound	Area	Final Conc	Unit
Multi24-08-27-Insekten-03.d	Multi-Mix-Std-N	Sample	7.087	Chloramphenicol	1050	560.4715	ng/g
Multi24-08-27-Insekten-04.d	LW+Z6 1:100	Cal	7.104	Chloramphenicol	8892	4744.0586	ng/g
Multi24-08-27-Insekten-05.d	LW+Z5 1:100	Cal	7.095	Chloramphenicol	5974	3187.3629	ng/g
Multi24-08-27-Insekten-06.d	LW+Z4 1:100	Cal	7.112	Chloramphenicol	4168	2223.7728	ng/g
Multi24-08-27-Insekten-07.d	LW+Z3 1:100	Cal	7.104	Chloramphenicol	2294	1223.9073	ng/g
Multi24-08-27-Insekten-08.d	LW+Z2 1:100	Cal	7.095	Chloramphenicol	1102	587.8189	ng/g
Multi24-08-27-Insekten-09.d	LW+Z1 1:100	Cal	7.112	Chloramphenicol	230	122.5590	ng/g
Multi24-08-27-Insekten-10.d	LW Mehlwürmer 1:100	Sample	7.087	Chloramphenicol	19	9.9670	ng/g
Multi24-08-27-Insekten-17.d	240382889A 1:100	Sample	7.104	Chloramphenicol	2380	1269.9303	ng/g
Multi24-08-27-Insekten-18.d	240382889B 1:100	Sample	7.095	Chloramphenicol	2861	1526.5922	ng/g
Multi24-08-27-Insekten-19.d	240382890A 1:100	Sample	7.087	Chloramphenicol	354	189.0354	ng/g
Multi24-08-27-Insekten-20.d	240382890B 1:100	Sample	7.095	Chloramphenicol	348	185.8752	ng/g
Multi24-08-27-Insekten-21.d	240382891A 1:100	Sample	7.197	Chloramphenicol	16	8.6393	ng/g
Multi24-08-27-Insekten-22.d	240382891B 1:100	Sample	7.172	Chloramphenicol	11	5.9262	ng/g

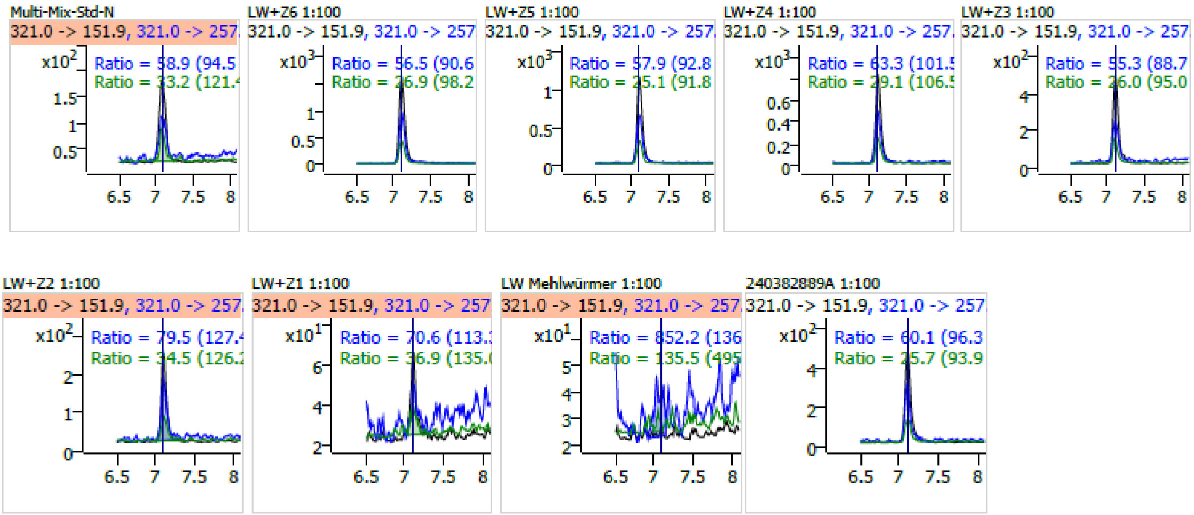

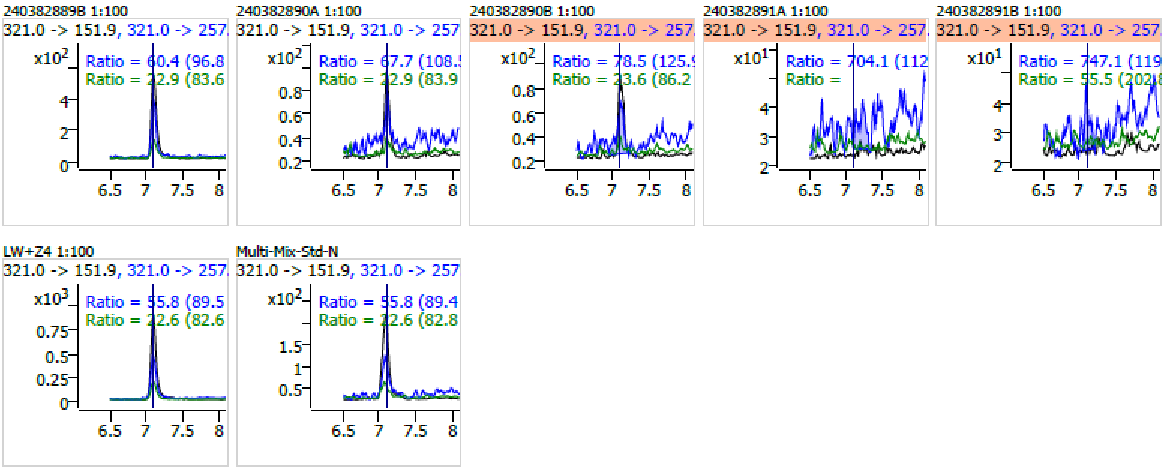

Erythromycin quantification in meal worms

Sample IDs Erythromycin:

240382876 : Meal worms after 0 h fasting
240382877 Meal worms after 24h fasting
240382878 5: Control

Screening

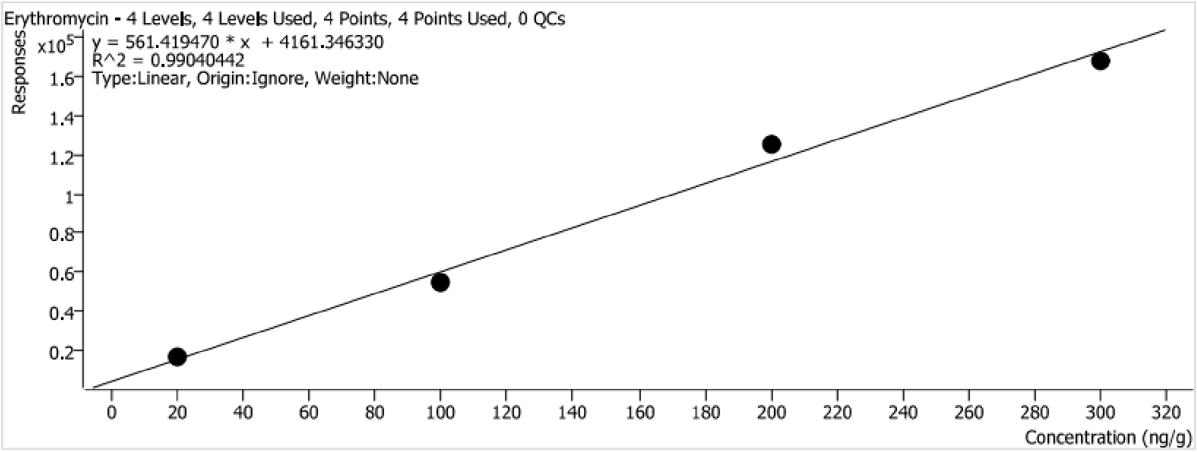

Quantifier

Data File	Sample Name	Compound	RT	Area	Final Conc	Unit
Multi24-08-14-M+Mehlwürmer-CAP-Erythr-03.d	Multi-Mix-Std-M	Erythromycin	7.953	11251711	20034.1287	ng/g
Multi24-08-14-M+Mehlwürmer-CAP-Erythr-29.d	LW MW+10ppb CAP+200ppb Erythromycin	Erythromycin	7.944	144421	249.8307	ng/g
Multi24-08-14-M+Mehlwürmer-CAP-Erythr-30.d	LW MW+10ppb CAP+200ppb Erythromycin	Erythromycin	7.944	148748	257.5383	ng/g
Multi24-08-14-M+Mehlwürmer-CAP-Erythr-31.d	LW MW+10ppb CAP+200ppb Erythromycin	Erythromycin	7.944	148451	257.0091	ng/g
Multi24-08-14-M+Mehlwürmer-CAP-Erythr-32.d	LW Mehlwürmer	Erythromycin	7.978	224	0.0000	ng/g
Multi24-08-14-M+Mehlwürmer-CAP-Erythr-33.d	LW Mehlwürmer	Erythromycin	7.894	73	0.0000	ng/g
Multi24-08-14-M+Mehlwürmer-CAP-Erythr-34.d	240382876 0h Erythr.	Erythromycin	7.910	16753236	29833.4407	ng/g
Multi24-08-14-M+Mehlwürmer-CAP-Erythr-35.d	240382877 24h Erythr.	Erythromycin	7.733	15774187	28089.5595	ng/g
Multi24-08-14-M+Mehlwürmer-CAP-Erythr-36.d	240382878 Kontrollgr-Erythr.	Erythromycin	7.944	11416	12.9212	ng/g
Multi24-08-14-M+Mehlwürmer-CAP-Erythr-37.d	240382889 0h CAP	Erythromycin	7.953	2817	0.0000	ng/g
Multi24-08-14-M+Mehlwürmer-CAP-Erythr-38.d	LW MW+10ppb CAP+200ppb Erythromycin	Erythromycin	7.953	136163	235.1216	ng/g
Multi24-08-14-M+Mehlwürmer-CAP-Erythr-39.d	Multi-Mix-Std-M	Erythromycin	7.953	9781312	17415.0543	ng/g

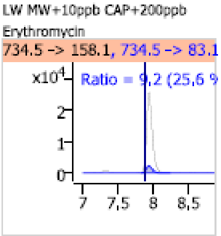

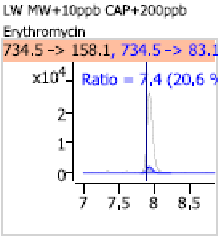

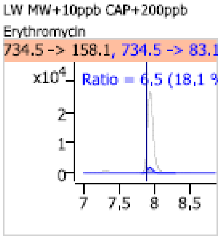

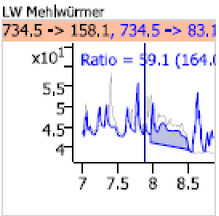

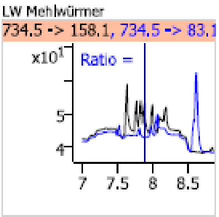

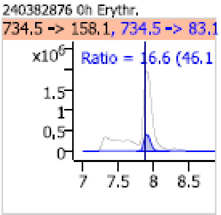

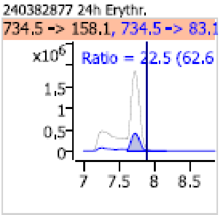

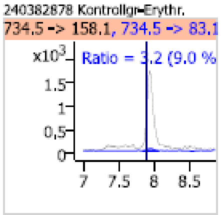

Erythromycin quantification 1:100

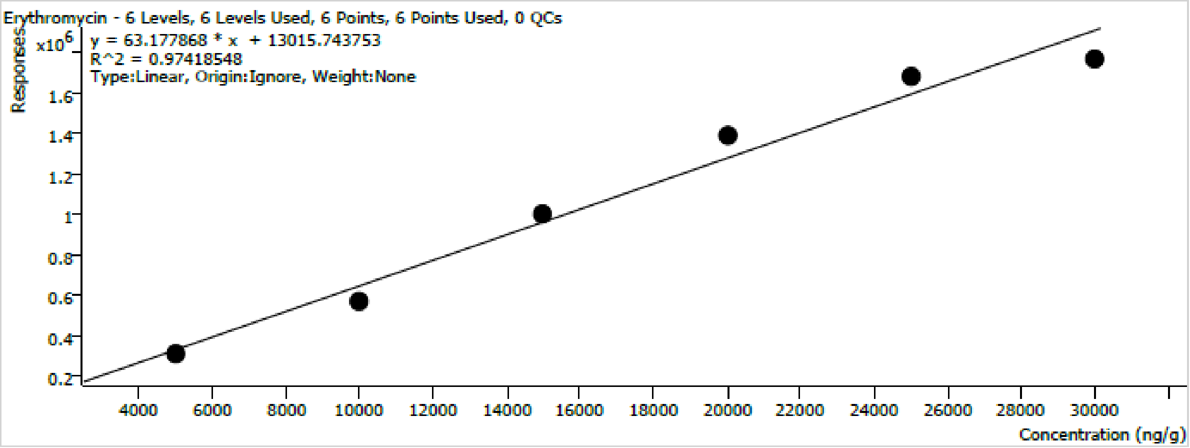

Quantifier

Data File	Sample Name	Sample Type	RT	Compound	Area	Final Conc	Unit
Multi24-08-27-Insekten-03.d	Multi-Mix-Std-N	Sample	7.944	Erythromycin	6775287	107035.4405	ng/g
Multi24-08-27-Insekten-04.d	LW+Z6 1:100	Cal	7.961	Erythromycin	1772028	27842.2147	ng/g
Multi24-08-27-Insekten-05.d	LW+Z5 1:100	Cal	7.961	Erythromycin	1678583	26363.1499	ng/g
Multi24-08-27-Insekten-06.d	LW+Z4 1:100	Cal	7.969	Erythromycin	1390469	21802.7853	ng/g
Multi24-08-27-Insekten-07.d	LW+Z3 1:100	Cal	7.961	Erythromycin	995477	15550.7236	ng/g
Multi24-08-27-Insekten-08.d	LW+Z2 1:100	Cal	7.961	Erythromycin	570657	8826.5234	ng/g
Multi24-08-27-Insekten-09.d	LW+Z1 1:100	Cal	7.969	Erythromycin	304557	4614.6030	ng/g
Multi24-08-27-Insekten-10.d	LW Mehlwürmer 1:100	Sample	7.969	Erythromycin	352	0.0000	ng/g
Multi24-08-27-Insekten-11.d	240382876A 1:100	Sample	7.961	Erythromycin	3585068	56539.6116	ng/g
Multi24-08-27-Insekten-12.d	240382876B 1:100	Sample	7.961	Erythromycin	6276707	99143.7560	ng/g
Multi24-08-27-Insekten-13.d	240382877A 1:100	Sample	7.961	Erythromycin	3501228	55212.5734	ng/g
Multi24-08-27-Insekten-14.d	240382877B 1:100	Sample	7.961	Erythromycin	3425535	54014.4782	ng/g
Multi24-08-27-Insekten-15.d	240382878A 1:100	Sample	7.953	Erythromycin	376	0.0000	ng/g
Multi24-08-27-Insekten-16.d	240382878B 1:100	Sample	7.792	Erythromycin	26	0.0000	ng/g

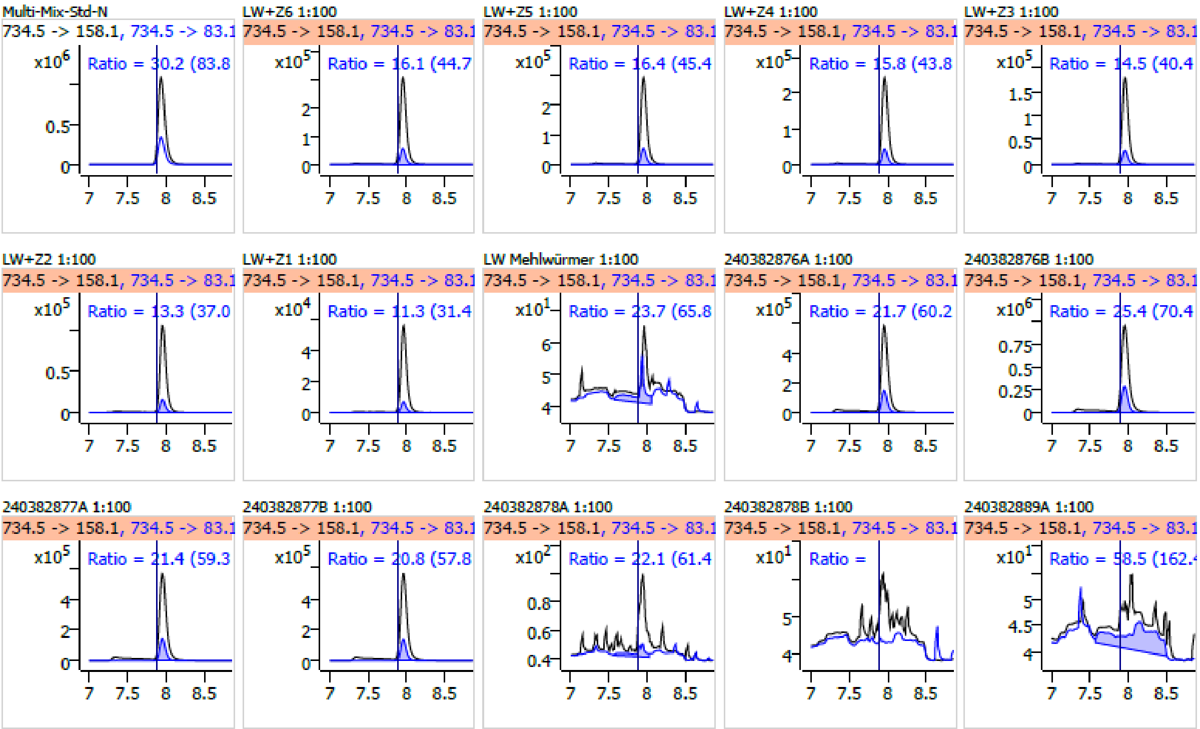

Erythromycin quantification 1:500

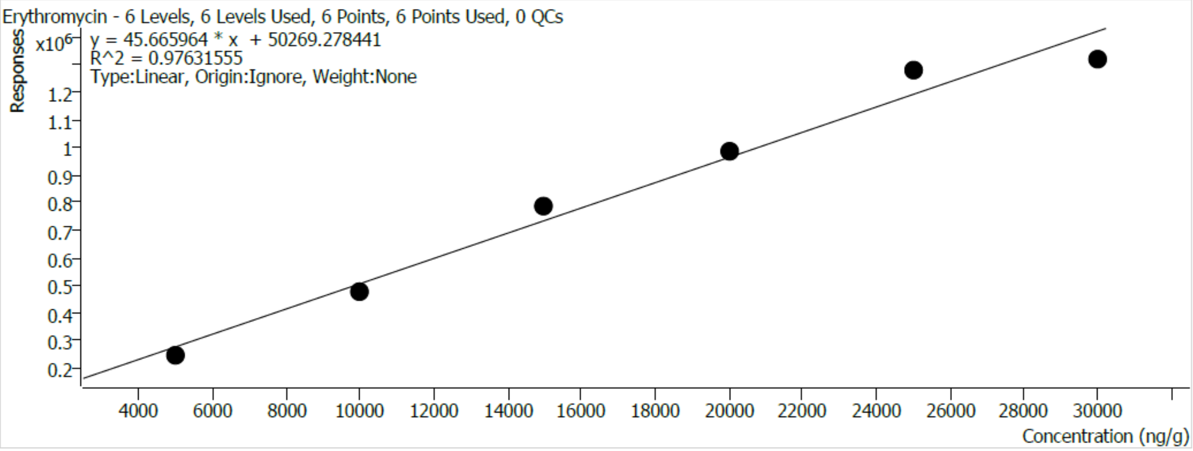

Quantifier

Data File	Sample Name	Sample Type	RT	Compound	Area	Final Conc	Unit
Multi24-08-29-Insekten-03.d	Multi-Mix-Std-N	Sample	7.978	Erythromycin	4898811	106174.0916	ng/g
Multi24-08-29-Insekten-04.d	LW MW+Z6 1:100	Cal	7.978	Erythromycin	1322878	27867.7679	ng/g
Multi24-08-29-Insekten-05.d	LW MW+Z5 1:100	Cal	7.969	Erythromycin	1280971	26950.0875	ng/g
Multi24-08-29-Insekten-06.d	LW MW+Z4 1:100	Cal	7.969	Erythromycin	982926	20423.4590	ng/g
Multi24-08-29-Insekten-07.d	LW MW+Z3 1:100	Cal	7.969	Erythromycin	785018	16089.6286	ng/g
Multi24-08-29-Insekten-08.d	LW MW+Z2 1:100	Cal	7.969	Erythromycin	480040	9411.1762	ng/g
Multi24-08-29-Insekten-09.d	LW MW+Z1 1:100	Cal	7.969	Erythromycin	244710	4257.8808	ng/g
Multi24-08-29-Insekten-10.d	LW Mehlwürmer 1:100	Sample	7.961	Erythromycin	265	0.0000	ng/g
Multi24-08-29-Insekten-11.d	240382876A 1:500	Sample	7.969	Erythromycin	1382237	29167.6355	ng/g
Multi24-08-29-Insekten-12.d	240382876B 1:500	Sample	7.969	Erythromycin	2821400	60682.6312	ng/g
Multi24-08-29-Insekten-13.d	240382877A 1:500	Sample	7.969	Erythromycin	1248537	26239.8362	ng/g
Multi24-08-29-Insekten-14.d	240382877B 1:500	Sample	7.969	Erythromycin	1233777	25916.6223	ng/g
Multi24-08-29-Insekten-15.d	240382878A 1:500	Sample	7.969	Erythromycin	451	0.0000	ng/g
Multi24-08-29-Insekten-16.d	240382878B 1:500	Sample	7.995	Erythromycin	315	0.0000	ng/g
Multi24-08-29-Insekten-17.d	LW MW+Z4 1:100	Sample	7.969	Erythromycin	1044342	21768.3405	ng/g
Multi24-08-29-Insekten-18.d	Multi-Mix-Std-N	Sample	7.961	Erythromycin	5636023	122317.6637	ng/g

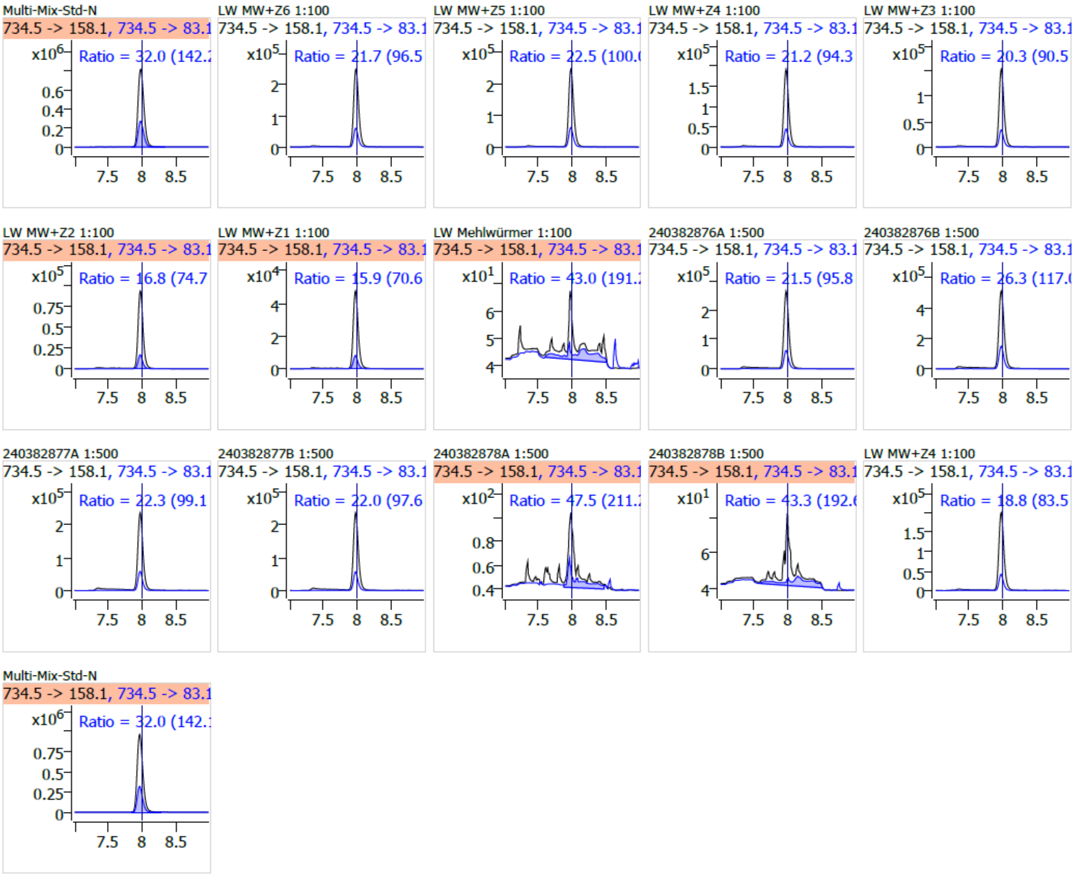
Multi-Mix-Std-N
734.5 -> 158.1, 734.5 -> 83.1
Ratio = 32.0 (142.
LW MW+Z6 1:100
734.5 -> 158.1, 734.5 -> 83.1
Ratio = 21.7 (96.5
LW MW+Z5 1:100
734.5 -> 158.1, 734.5 -> 83.1
Ratio = 22.5 (100.
LW MW+Z4 1:100
734.5 -> 158.1, 734.5 -> 83.1
Ratio = 21.2 (94.3
LW MW+Z3 1:100
734.5 -> 158.1, 734.5 -> 83.1
Ratio = 20.3 (90.5
LW MW+Z2 1:100
734.5 -> 158.1, 734.5 -> 83.1
Ratio = 16.8 (74.7
LW MW+Z1 1:100
734.5 -> 158.1, 734.5 -> 83.1
Ratio = 15.9 (70.6
LW Mehlwürmer 1:100
734.5 -> 158.1, 734.5 -> 83.1
Ratio = 43.0 (191.
240382876A 1:500
734.5 -> 158.1, 734.5 -> 83.1
Ratio = 21.5 (95.8
240382876B 1:500
734.5 -> 158.1, 734.5 -> 83.1
Ratio = 26.3 (117.
240382877A 1:500
734.5 -> 158.1, 734.5 -> 83.1
Ratio = 22.3 (99.1
240382877B 1:500
734.5 -> 158.1, 734.5 -> 83.1
Ratio = 22.0 (97.6
240382878A 1:500
734.5 -> 158.1, 734.5 -> 83.1
Ratio = 47.5 (211.
240382878B 1:500
734.5 -> 158.1, 734.5 -> 83.1
Ratio = 43.3 (192.
LW MW+Z4 1:100
734.5 -> 158.1, 734.5 -> 83.1
Ratio = 18.8 (83.5
Multi-Mix-Std-N
734.5 -> 158.1, 734.5 -> 83.1
Ratio = 32.0 (142.
